# Temperature regulation in horses during exercise and recovery in a cool environment

**DOI:** 10.1186/1751-0147-54-42

**Published:** 2012-07-17

**Authors:** Hanna Wallsten, Kerstin Olsson, Kristina Dahlborn

**Affiliations:** 1 Department of Anatomy, Physiology, and Biochemistry, Faculty of Veterinary Medicine and Animal Science, Swedish University of Agricultural Sciences, Box 7011, SE-750 07, Uppsala, Sweden

**Keywords:** Cool ambient temperature, Heart rate, Rectal temperature, Respiratory rate, Skin temperature

## Abstract

**Background:**

Clipping the winter coat in horses is done to improve heat dissipation during exercise and make grooming easier. It is often combined with blanketing to keep the horse warm. The aims of the present study were to investigate how clipping and the use of blankets affect thermoregulation during exercise and recovery in horses.

**Methods:**

One Gotland pony, one New Forest pony, and one warm-blooded horse exercised one after the other on a 6450 m long track. The horses walked, trotted and cantered according to a predetermined scheme, which took about 50 minutes including three stops. The scheme was repeated on five consecutive days when horses were: 1) unclipped 2) unclipped + blanket during recovery, 3) left or right side clipped, 4) clipped, and 5) clipped + riding blanket + blanket during recovery. Heart rate (HR) was measured with telemetry, respiratory rate (RR) by counting flank contractions, skin temperatures by thermistor probes, and rectal temperature with a digital thermometer. Skin wetness (SW) was estimated by ocular inspection (dripping = 5, dry = 0).

**Results:**

Mean outdoor temperature varied from -1.1 to - 8.7°C. HR increased progressively during exercise with no difference between treatments. Maximum RR was 77 ± 30 breaths/min (unclipped) and 49 ± 27 breaths/min (clipped). The lowest skin temperature was 17.5 ± 2.7°C in a hind leg during exercise, which increased to 34.5 ± 0.1°C during recovery. Rectal temperature was elevated during recovery in unclipped, but not in clipped horses and skin temperature at base of tail was elevated during recovery except in unclipped horses without blanket. Moisture after exercise scored 3.2 ± 0.8 in unclipped and zero in clipped horses.

**Discussion and conclusion:**

Leg skin temperature initially dropped at onset of exercise in clipped horses, and then increased after about 30 minutes due to internal heat from the working muscles. These changes were not significant when clipped horses had riding blankets, whereas unclipped horses became overheated as judged from respiratory rate and elevated rectal temperature. Providing clipped horses with blankets dampened the changes in leg skin temperature during exercise.

## Background

Core temperature in horses is normally regulated within narrow limits. Information from thermal receptors in the skin and internal organs are integrated in hypothalamus, which is the dominant controller over body temperature [1,2]. Skin blood flow can vary from almost zero to one third of cardiac output by opening and closing arterio-venous shunts or precapillary vessels. It approaches zero in cool weather by maximal vasoconstriction mediated by adrenergic sympathetic nerves, whereas sympathetic vasodilator nerves are activated during hyperthermia and are responsible for most of the vasodilatation that occur [3,4].

Horses are found at ambient temperatures from – 40 to 40°C. Depending on management routines, breed and activities horses may grow a thick winter coat [5,6]. In cool-acclimated horses at rest the lower critical temperature of the environment has been reported to be – 15°C [5]. In horses kept in stables at night and not acclimatized to winter climate the lower critical temperature has been estimated to 5°C [7].

Horses have a high metabolic capacity combined with a small surface area for dissipation of heat. When horses exercise about 20% of the metabolism in the muscle cells is used for work and the remaining 80% becomes heat [8]. Skin blood flow increases during internal body heating transferring the heat from body core to the surface of the skin and if vasodilatation is not sufficient sweating starts [3].

Horses adapted to cool winter climate exercising in a climatic chamber at temperatures ranging from 5 to 18°C showed more efficient evaporation after they had been clipped than comparable experiment with winter coat intact [9]. Horses that compete during winter time may thus benefit from clipping, but blanketing at rest may be needed to keep the clipped horse warm [10].

Comparatively few studies have studied cool-acclimated horses exercising outdoors at ambient temperatures below zero [7,11]. We hypothesized that horses with winter coats benefit from clipping by mobilizing more efficient heat dissipation during exercise and thereby not become overheated. A thick winter coat limits transfer of heat from the body surface by convention because the air is trapped in the fur, and sweating is also less efficient since sweat wets the fur instead of being directly evaporated from the skin [12].

The aims of the present study were to investigate how clipping the winter fur changes skin and rectal temperatures, respiratory rate, and sweating in horses exercising in the field at an ambient temperature below zero and during recovery in the unheated stable. Experiments were conducted both with and without blankets.

## Materials and methods

### Animals

One New Forest pony, mare, age 14 years, body weight 450 kg, one Gotland pony, castrated male, 24 years old, body weight 280 kg, and a riding horse, mixed breed, castrated male, 11 years old, body weight 550 kg were studied. They were routinely fed in the stable and then let out in a corral at about 08.00 h. During normal routines the horses spent all day outdoors until about 16.00 h. On cool days and at night they were provided with blankets.

On experimental days the horses were fed at about 07.30 h and kept indoors. Control measurements started at about 09.30 h and each day the horses were exercised in the following order: 1) Riding horse 2) New Forest pony and 3) Gotland pony.

The study was performed outside Uppsala, Sweden in December 2009. The local Ethical Committee, Uppsala, Sweden approved the experimental design.

### Experimental procedures

In the morning of an experimental day temperature and humidity were measured with a thermo-hygrometer (Biltema, Uppsala, Sweden) and the weather was noted (Table 1).

**Table 1:** Weather on the days when three horses, subjected to different treatments on five consecutive days, were exercising outdoors

**Day**	**Treatment**	**Temperature (°C)**		Relative humidity (%), precipitation*	Wind speed (m/sec), direction*
		**Indoor**	**Outdoor**		
1	**Unclipped**	1.6	– 2.0 ± 0.2	99 ± 0; Light snowing	3 ± 2, east
2	**Unclipped, Blanket**^**#**^	2.2	– 6.4 ± 0.6	95 ± 2; Irregular snowing	0 ± 1, southeast
3	**Half-clipped**	-	−1.1 ± 0.4	99 ± 0; Rain-snow	2.5 ± 1, south
4	**Clipped**	2.8	– 6.9 ± 0.2	91 ± 1; Irregular snowing	1 ± 1, northwest
5	**Clipped, Blankets**^**##**^	0.6	– 8.7 ± 1.7	91 ± 6; Light snowing	1.7 ± 1, north

Mean ± SD. *Courtesy of Swedish Meteorological and Hydrological Institute, Sweden.

# fleece blanket after exercise, ##, riding blanket and fleece blanket after exercise.

Small roads near the stable were used as a cross country track for exercise. The track measured 6450 m and was snow-covered on all days. Two of the horses were ridden and the Gotland pony was driven by one of the authors. After control measurements in the stable, horses did four exercise sessions and a short walk according to a pre-determined schedule interrupted by pauses for measurements (Table 2). The experiment ended with measurements every 5^th^ min for 30 min during recovery (Table 2). The same procedure was repeated on five days.

**Table 2:** Protocol followed by three horses when exercising outdoors one after the other on five consecutive days

**Event**	**Sample**	**Measurements**
Before, in stable	1 and 2	HR, RR, RT, ST , STt*
*Session I: Walk, 800m + Trot, 1200 m*		
STOP	3	HR, RR, ST
	4 (after ca 2 min)	HR
*Session II: Trot 400 m + Canter 1000 m*		
STOP	5	HR, RR, ST
	6 (after ca 2 min)	HR
*Session III: Canter, 1200 m*		
STOP	7	HR, RR, ST
	8 (after ca 2 min)	HR
*Session IV: Canter, 600 m + Trot, 200 m + Walk 200m + Canter 150 m*		
**(No stop)**	9	HR
*Session V: Walk, 700 m*		
Recovery, stable	10 ­ 16	HR, RR, RT, ST , STt

*HR = heart rate, RR = respiratory rate, RT = rectal temperature,

ST = skin temperatures on hind legs and the neck, STt = tail skin temperature.

The treatments were: 1) Intact winter fur (=unclipped) 2), Intact winter fur + blanket (fleece material) during recovery, 3) left or right side clipped, 4) both sides clipped, 5) clipped + riding blanket + fleece blanket during recovery. The fifth day was the coolest day and driving the Gotland pony was therefore done the next day. One of the horses was randomly picked to be provided with a blanket on the first day and the other two got no blanket. Next day these two horses were provided with blankets. Likewise, which side would be shaved first was picked at random and two of the horses were first shaved on their left side.

During recovery the horses were tied up in the stable and measurements were done every 5^th^ min for 30 minutes (Table 2). The indoor temperature varied between 0.6 and 2.8°C (Table 1).

### Equipment

Heart rate was measured telemetrically (Polar Equine CS600 Trotting Monitor) with the sensor body (Polar WearLink® W.I.N.D) fastened at the level of the heart with a belt secured with two layers of Vet-flex bandage placed in front of the saddle-girth. The signals were transmitted to a receiver in a modified wrist-watch during the exercise. Data were registered every 15 second and transferred to the computer after end of experiment (Polar Pro Trainer 5). Respiratory frequency was measured by counting the flank movements during 30 seconds. Rectal temperature was measured with a digital thermometer.

Skin temperatures were measured by pressing probes with sensors against the skin (ELLAB, Rödovre, Danmark). The equipment senses temperatures between – 1 and 50 °C. Skin temperatures were measured on left and right side of the neck (halfway from head to withers). Two of the horses had the mane on the right side and one on the left and the probe was held just under the mane. On the legs the probe was pressed against the skin at a point on the anterior border of bicepts femoris, halfway from spine to knee and finally on a spot on the back just in front of the tail base (left unclipped) as a reference point (=“tail skin temperature”). At each spot the skin temperature was measured during 30 seconds and in the order neck on left side, left hind leg, neck right side, right hind leg, and tail.

After exercise the sweating was estimated. The scale was 0 = dry and 5 = sweat drops clearly visible.

### Statistical analyses

Values are presented as means and standard deviation (SD). The data were analyzed with SAS® software (SAS Institute, Cary, NC, USA). The repeated measurement analysis of variance (using the MIXED procedure) was applied to the parameters. The statistical model included event, sample, the interaction between event and sample, and the random effect of horse. Least square means were estimated and pair-wise test of significance were performed for the differences between the estimated means. Bonferroni adjustment was used to limit the risk for false mass significance. The significance level was set at p ≤ 0.05.

## Results

Indoor and outdoor temperatures and weather conditions are found in Table 1.

### Heart rate

**Figure 1:**
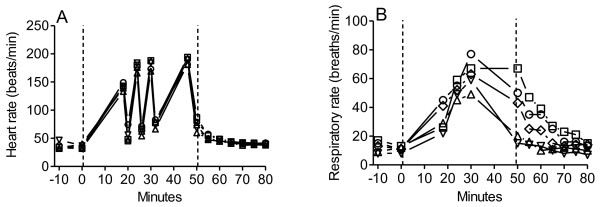
**Heart and respiratory rates in three horses before and during exercise and recovery**. Horses exercised one after the other on a 6450 m long track during five consecutive days according to a pre-determined schedule which included four exercise sessions interrupted by pauses. Treatments were: unclipped (circles), unclipped with blanket during recovery (square), half-clipped (romb), clipped (triangle pointing upwards), and clipped with riding blanket and blanket during recovery (triangle pointing downwards). Hatched lines show onset and end of exercise. **A**) Heart rate increased at each run (P < 0.001 *vs*.baseline), and decreased at each pause, but remained elevated (p < 0.01 - 0.001). **B**) Respiratory rate increased in all treatments (p < 0.05 - 0.001). RR remained elevated in unclipped horses with blanket upon return to the stable and it was higher than in clipped horses (p < 0.001) at that time.

HR increased during all exercise sessions and decreased at each stop with no difference between treatments as illustrated in Figure 1A. In the statistical analysis values from all treatments were therefore pooled. On average HR was 36 ± 7 beats/min before exercise. After the first run it was 141 ± 11 (p < 0.001). It increased further to 176 ± 12, to 181 ± 14, and to 188 ± 10 beats/min at the exercise sessions (p < 0.001 vs. before exercise for all). At the first stop HR was 53 ± 16 (p < 0.01 vs. before exercise), at the second stop 63 ± 16 (*p* < 0.001), and at the third stop 75 ± 14 beats/min (*p* < 0.001), respectively. It took HR 15 min after the last run to return to basal levels.

### Respiratory frequency

RR increased from 13 ± 6 to 77 ± 30 breaths/min (p < 0.001) when the horses were unclipped, and to 67 ± 20 breaths/min (p < 0.001) when unclipped with blanket. When the horses were clipped RR was 11 ± 5 breaths/min before exercise and increased to 49 ± 27 (p < 0.05) and to 59 ± 26 breaths/min (p < 0.05) without and with blanket, respectively. RR decreased immediately after the last exercise in all treatments except when they were unclipped with blanket. In that experiment the highest value during exercise prevailed during the last walk to the stable (Figure 1B).

### Rectal temperature and tail skin temperature

Rectal temperature before exercise was 37.4 ± 0.2 and during recovery 38.0 ± 0.3°C (p < 0.01) or 38.2 ± 0.6°C (p < 0.001) when the horses were unclipped or unclipped with blankets, respectively (Table 3). During the other treatments rectal temperature was not significantly elevated after exercise.

**Table 3:** Rectal and tail skin temperatures in three horses subjected to different treatments on five consecutive days and exercising outdoors

**Treatment**	**Rectal temperature °C**		**Skin tail temperature °C**	
	**Before**	**Recovery**	**Before**	**Recovery**
Unclipped	37.4 ± 0.2	38.0 ± 0.3*	30.9 ± 1.6	32.9 ± 2.4
Unclipped^1^	37.4 ± 0.2	38.2 ± 0.6*	31.1 ± 1.0	34.5 ± 1.2*
Half-clipped	37.5 ± 0.2	38.1 ± 0.4	30.4 ± 1.1	33.4 ± 0.9*
Clipped	37.6 ± 0.2	37.8 ± 0.4	29.6 ± 1.2	32.1 ± 1.8*^#^
Clipped^2^	37.6 ± 0.1	37.8 ± 0.3	29.7 ± 0.5	33.5 ± 1.3*

Mean ± SD of two samples before and six samples after (recovery) exercise.

^1^ Winter coat and blanket during recovery.

^2^ Coat clipped and riding blanket followed by blanket during recovery.

*p < 0.05 – 0.001 vs. before exercise, and ^#^ p < 0.05 -0.01 vs. treatment “Unclipped”.

Tail skin temperature was elevated after exercise with the exception of the first treatment (Table 3). Before exercise the highest temperature was 31.1 ± 1.0°C (clipped with blanket) and lowest 29.6 ± 1.2°C (unclipped horses). Mean values after exercise were 34.5 ± 1.2°C (p < 0.001) and 32.1 ± 1.8°C (p < 0.05), respectively. Also when horses were half-clipped or clipped with blankets the tail skin temperature was elevated after exercise (Table 3).

### Skin temperature on neck and hindlegs

**Figure 2:**
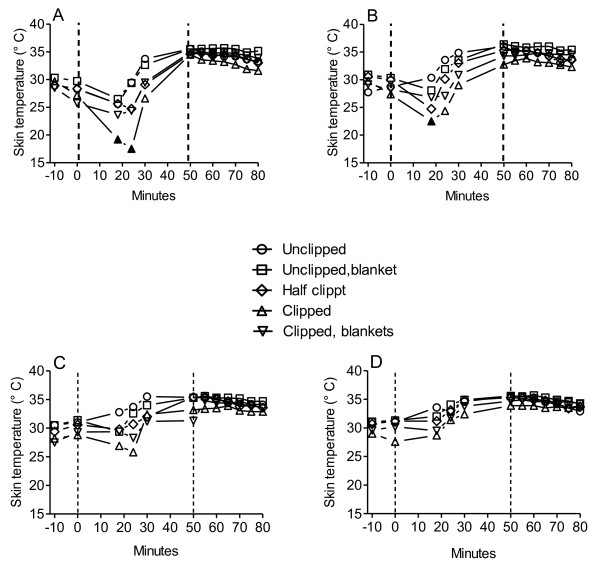
**Skin temperatures before, during and after exercise in three horses. A:** Skin temperatures on left hind leg and **B:** right hind leg, **C:** Skin temperature on the neck side with no mane and **D:** opposite neck side with mane. Horses exercised one after the other on a 6450 m long track during five consecutive days according to a pre-determined schedule which included four exercise sessions interrupted by pauses. Hatched lines show onset and end of exercise. Symbols written in the center of figure represent one of each treatments, respectively, and when filled p < 0.05.

Skin temperatures changed when the horses came out in the cold and started to exercise (Figure 2). Skin temperature in the left hind leg decreased from 28.4 ± 1.8°C to a minimum of 17.5 ± 27°C ( p < 0.001) and on the right hind leg from 28.6 ± 1.7 to 24.4 ± 4.1°C ( p < 0.01), respectively when the horses were clipped (Figure 2A and Figure 2B, respectively). It increased during recovery to 34.5 ± 0.1 (p < 0.001 vs. exercise) and 32.7 ± 1.2°C (p < 0.001 vs. exercise), respectively.

When left leg temperature changes were summarized before, during, and after exercise it did not change during exercise as long as horses were unclipped or half-clipped, but fell when horses were clipped and clipped with blankets. Upon recovery skin temperature became elevated above pre-exercise values on all days (p < 0.01-0.001). In the right hind leg summarizing the temperatures revealed a drop during exercise in clipped horses with blankets and upon recovery skin temperatures became elevated on all treatments (p < 0.01 -0.001).

In Figure 2C and in Figure 2D neck skin temperature on the side without mane and with mane, respectively is shown. Neck skin temperature did not change significantly during exercise, but during recovery it was higher than before exercise on both sides of the neck and in all treatments.

### Skin moisture

When horses returned to the stable, moisture scored 3.2 ± 0.8 when unclipped and 3.7 ± 1.5 when unclipped with blanket. When half clipped moisture scored 2.0 ± 1.0 on the unclipped side and 0.0 on the clipped side. When fully clipped all horses scored zero.

## Discussion

Despite different breeds, age and sex the three horses reacted similarly to the exercise challenge in the cold. The results illustrate how the cool environment first mobilized cool defence mechanisms and how the intensity, duration and frequency of exercise made the regulatory mechanisms to switch to heat dissipation.

The muscles demand increased blood flow during exercise, which is achieved by both a rise in heart rate and stroke volume. HR was measured and it increased during each exercise session with no difference between horses. It indicates that the intensity of exercise was similar, which made comparison between the responses of the horses to treatments meaningful.

In resting horses limb surface temperatures vary in direct proportion to changes in ambient temperature between 5 to 25°C [13]. In cool conditions the autonomic nervous system directs blood away from the skin surface on extremities to internal organs and skin temperature falls. Peripheral vasoconstriction and closing of arterio-venous shunts are mediated by the sympathetic noradrenergic vasoconstrictor nerves and represents the first defense reaction during exposure to a cool environment [3,14].

In the present study the first exercise session consisted of walking and trotting and skin temperature in the legs dropped in clipped horses. The reaction could have been initiated by a reflex vasoconstriction in the skin vessels at the onset of exercise [15], but in this study the drop in skin temperature was observed after about 20 minutes of exercise. It appears that the effect was due to a combination of the low environmental temperature and the low intensity of exercise so that the muscles did not produce enough heat to raise core temperature. Therefore, heat dissipation mechanisms were not mobilized, and more blood could go to the muscles instead of to the skin for vasodilatation. However, as intensity of each exercise session increased the heat produced by the working muscles overcame the influence from the cool environment and skin temperature in the legs increased above basal level during recovery in all treatments.

There is some controversy whether a combination of exercise and environmental heat results in a competition between the exercising skeletal muscle and the skin compartments for the available blood flow [15]. In the present study blood flow to the skin was markedly different on the neck and legs as judged from skin temperature. The low skin temperature in the legs could partly have been due to competition from the leg muscles which worked much more than neck muscles.

As the horses continued to exercise, the muscle work created more internal body heat, which elicited reflex neurogenic vasodilatation in the skin resulting in increased skin temperatures in legs as well as in the neck. In all treatments skin temperatures increased at the end of exercise, even in clipped horses, showing the efficient heat production of exercising muscles.

Unclipped horses had a wet hair coat after exercise, but no moisture could be observed in clipped horses. It is possible that sweat rapidly evaporated from the naked skin thereby efficiently preventing overheating due to exercise. It could explain the unchanged rectal temperature and lower respiratory frequency in clipped horses. However, sweat rate was not measured and it can therefore not be excluded that non-evaporative heat dissipation in the cool ambient temperature was sufficient to prevent an increased rectal temperature. Whichever the explanation, the horses seemed to benefit from clipping since they did not need to mobilize heat dissipating mechanisms.

The results show that the physiological thermoregulatory processes were sufficient to maintain basal body temperature in clipped horses during exercise in the cold and when the horse was stabled afterwards at a temperature near zero. Horses with winter coat showed small tendencies to peripheral vasoconstriction. Instead skin temperatures increased soon after onset of exercise and also rectal temperature increased. Similar results were found before and after clipping horses during exercise and recovery in a climatic chamber, although those experiments were performed at an ambient temperatures above zero [9].

No ill effects were noticed in these horses during or after exercising in the cold, which was expected based on the thorough investigations of reactions of horses exercising at – 25°C [ 11].

### Study limitations

The number of horses was few. However, they had been kept by the same management and routines for a long time. The horses did the exercises in the same order. Weather conditions could change during the day, but no dramatic changes occurred in the present study. It cannot be taken for granted that the thermistor was not affected by the wind outdoors.

## Conclusions

Clipped horses exercising at outdoor temperature down to ~ 9°C below zero maintained rectal temperature. Respiratory rate took longer to return to basal levels after the exercise in horses with intact winter fur and blankets. Skin temperature is dependent on where it is measured on the body. Leg skin temperature can be useful for estimating when the horse has warmed up sufficiently for the skin vessels to vasodilatate. These results demonstrate that heat dissipating thermoregulatory mechanisms were mobilized in unclipped horses and cold-defense mechanisms at the onset of exercise in clipped horses. Blankets augmented heat dissipation in unclipped horses and dampened the responses in clipped horses. The mechanisms are physiological and energy-demanding and if horses lose water and electrolytes by sweating and increased evaporation from lungs and skin the fluid balance needs to be considered.

## Competing interests

All authors declare that they have no competing interests.

## Authors’ contributions

Hanna Wallsten took part in planning the studies, took care of the horses and was responsible for exercising them according to protocol, summarized data and drafted figures. Kristina Dahlborn planned the studies, was responsible for the horses and did all measurements. Kerstin Olsson assisted during the experiments, did the statistical analyses and finalized figures and tables. All authors participated in writing the manuscript.
